# Use of Himplant® for correction of residual deformity following prior treatment of peyronie’s disease: a case series

**DOI:** 10.1038/s41443-024-00840-7

**Published:** 2024-02-23

**Authors:** Laurence A. Levine, Elsayed Desouky, James J. Elist, Daniel Moon, Steven K. Wilson

**Affiliations:** 1 Department of Urology, RUSH University, Chicago, IL USA; 2 Emeritus, Department of Urology, Cedars-Sinai Medical Center, Los Angeles, CA USA; 3 A Medical Corporation, Beverly Hills, CA USA; 4 Institute for Urologic Excellence, La Quinta, CA USA

**Keywords:** Sexual dysfunction, Quality of life

## Abstract

Over the years, numerous non-surgical and surgical treatment options have been explored for Peyronie’s disease. Current options may result in incomplete correction of the deformity, which can be bothersome to the patient. This is a two-center case series of three patients who had previously undergone treatment for Peyronie’s disease. Patient 1 underwent plication with extratunical grafting. Patient 2 underwent a series of Xiaflex® injections and then subsequent surgical plication for residual curvature. Patient 3 underwent a series of Xiaflex® injections. The Himplant® subcutaneous silicone penile prosthesis was placed in a standardized manner through a scrotal incision in all cases to mask residual penile deformities and enhance penile girth after Peyronie’s disease treatment. Patients were contacted and asked 18 questions regarding satisfaction and erectile function with the responses recorded. This pilot study presents findings of high patient satisfaction, increases in flaccid penile length and girth, and an acceptable profile of adverse events following Himplant® placement. Based on our limited experience, we would consider offering Himplant® implantation when residual curvature is <40° and the penile indentation does not cause instability/buckling during penetrative sexual activity. Further research and larger studies are warranted to validate these findings and assess long-term outcomes and patient-reported satisfaction.

## Introduction

Peyronie’s disease (PD) is a scarring disorder of the penis causing penile deformity secondary to the formation of fibrotic tissue within the tunica albuginea [1, 2]. It is characterized by pain, the presence of palpable plaque/nodules, and deformity of the erect penis, including curvature and indentation, shortening. The etiology and pathophysiology of PD are hypothesized to result from penile trauma, atypical wound healing, and deposition of fibrotic tissue that does not undergo normal remodeling [3–5]. Suggested underlying risk factors include diabetes mellitus, Dupuytren’s contracture, and radical prostatectomy [6–8]. PD prevalence could be as high as 11% in the United States [9] and is likely underreported [10].

Treatment options include oral therapies, topical treatments, intralesional injection therapy including collagenase *Clostridium histolyticum* (Xiaflex®, Auxilium Pharmaceuticals Inc., Chesterbrook, PA, USA), and surgery [1, 2]. Several patients with PD who had undergone injection and/or surgery presented to our clinics with bothersome residual deformities. This case series presents Himplant® (International Medical Devices Inc., Los Angeles, CA, USA) as a surgical treatment option for patients to mask residual deformities and to enhance penile girth post-PD treatment.

Penuma® received 510 K clearance from the US Food and Drug Administration (FDA) in 2004 for the cosmetic correction of soft tissue penile deformities [11] and for cosmetic enhancement of the penis in May 2022 [12]. The device has primarily been used for potent men to enhance the flaccid and erect girth of the penis and to the enhance the length of the visible flaccid penis [13, 14]. A previous study found that the implant was a safe option for augmenting the flaccid penile dimensions in individuals experiencing penile aesthetic deformities. Complications mainly pertain to patient’s cosmetic concerns, which can be readily addressed [13]. Careful patient selection is of utmost importance to guarantee that patient expectations remain realistic and that the procedures yield successful results [15, 16].

In 2023, the Penuma® implant underwent design enhancement. This updated version, Himplant®, incorporates a crucial improvement over its predecessor; the mesh that was formerly attached externally for securing the implant to the distal penile shaft is now integrated into the Himplant® structure itself. The preoperative procedures including the criteria for patient selection, as well as the postoperative recovery protocols, remain consistent with the original Penuma® implant.

## Subjects and methods

This study comprises a case series involving three patients, focusing on the insertion of Himplant® for concealing penile deformities and for augmenting the flaccid length and girth of the penis. Selection criteria for participants included a documented history of PD, prior treatment for PD with Xiaflex® and/or surgery, with persistent penile deformities post-treatment, and a history of circumcision. Patients experiencing penile indentation that led to instability or buckling during sexual intercourse and those with residual penile curvature exceeding 40 degrees were excluded. Preoperative and postoperative physical examinations were conducted to evaluate the characteristics of penile deformity and to measure dorsal penile girth and midshaft length with a paper ruler. Patients 1 and 3 opted out of having their photographs taken for record. Patient 2 consented to the dissemination of photographic material as part of this study.

We received general Institutional Review Board (IRB) approval for reporting outcomes associated with Penuma®/Himplant® procedures. However, this approval does not extend specifically to specialized subgroup analyses, such as the one conducted with this cohort. We believe that our existing general IRB approval sufficiently encompasses the patient population under study.

### Case 1

Patient 1, a 54-year-old man, was diagnosed with PD in 2019 at the age of 52 years. At initial presentation, he had an estimated 45° dorsal and mild left lateral curvature with midshaft narrowing but no hinging. He used oral phosphodiesterase-5 inhibitors (PDE-5i) medications to augment his erections and sought treatment because of the bothersome curvature but observed no substantial benefits from traction therapy. Duplex ultrasound revealed a strong erection, a 50° dorsal and 20° left curve with mid-shaft narrowing, and no hinge effect. In 2021, he underwent tunica albuginea plication with extratunical grafting using a porcine dermal graft to correct the curvature and indentation. He reported satisfactory resolution of curvature and indentation correction but was dissatisfied with his loss of penile volume and girth that resulted from the plication surgery. Upon presentation at our clinic in 2022, examination of the patient’s penis revealed only narrowing of the penile shaft, but no curvature or indentation.

### Case 2

**Fig. 1:**
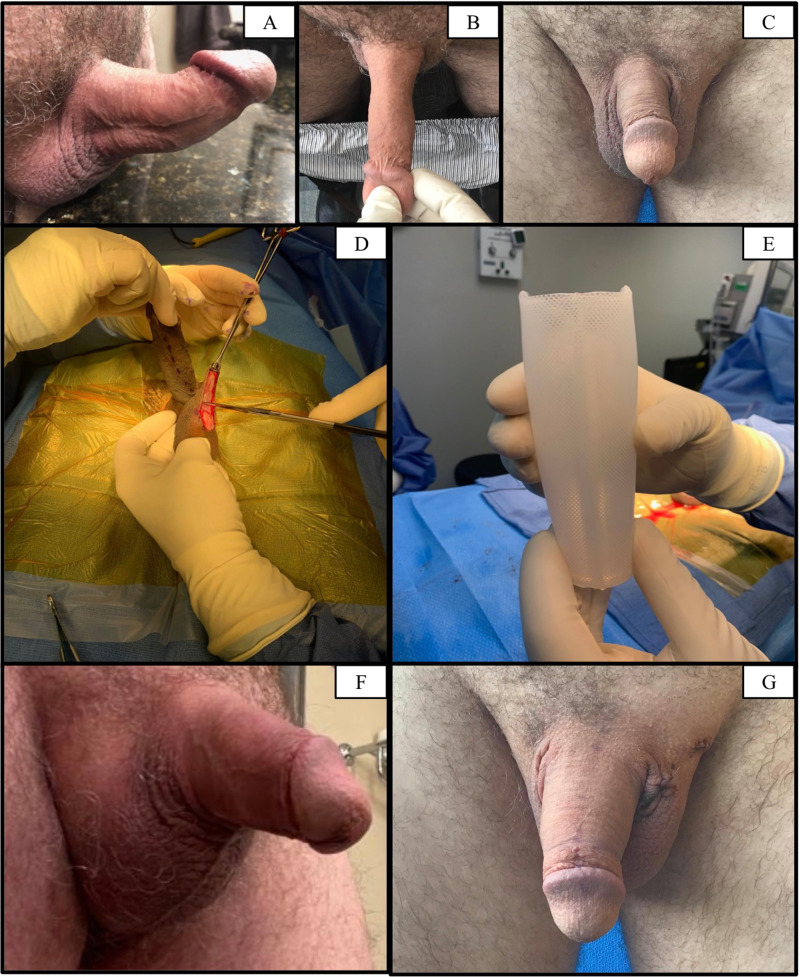
[Patient 2] Pre- and post-operative images; Himplant®. **A** Preoperative image; right view of erect penis; 40° dorsal and 20° left curvature. **B** Preoperative image; dorsal view of flaccid penis; proximal shaft narrowing and hourglass deformity. **C** Preoperative image; front view of flaccid penis; date of surgery. **D** High scrotal incision for Himplant®. **E** Himplant®; 16 cm length. **F** Postoperative image; right view of erect penis; correction of curvature. **G** Postoperative image; front view of flaccid penis; 3 days post-op.

Patient 2, a 64-year-old man with a history of chronic hypertension, hyperlipidemia, and male hypogonadism, was diagnosed with PD in 2014 at the age of 55. His degree of penile curvature prior to PD treatment was not recorded. He received three injections (0.9 mg) of Xiaflex® between 2014 and 2015 and underwent surgical plication in 2016. He reported satisfactory correction of curvature immediately post-operation but was bothered by loss of penile length. Over the next 4 years, the patient reported progressive recurrence of curvature and proximal shaft narrowing. Upon presentation to our clinic in 2022, examination of the patient’s erect penis revealed an estimated 40° dorsal and 20° left curvature with proximal shaft narrowing and a left lateral indentation but no hinging (Fig. 1A–C).

### Case 3

Patient 3, a 63-year-old man, was diagnosed with PD in 2017 at the age of 57. Evaluation of the erect penis revealed a 55° dorsal and 25° right curvature, reduced length (estimated 1.5 inches by the patient), and hinging. He received oral PDE-5i and four Xiaflex® injections (0.58 mg) in 2018. He subsequently reported satisfactory correction of the hinging and improved sexual function. However, over the next 15 months, he experienced progressive erectile dysfunction (ED), persistent bothersome curvature, and midshaft narrowing. Upon presentation at our clinic in 2022, examination of the patient’s erect penis revealed an estimated 30° dorsal and mild right curvature with midshaft narrowing but no hinging. This patient was offered an inflatable penile prosthesis since he had ED requiring oral therapy, which was worsening. He preferred to try the Himplant® first.

After undergoing their initial treatment for PD, all patients complained of mild residual deformity (curvature, indentation, and narrowing) that negatively affected their quality of life. The procedure for Himplant® placement to correct deformities and to provide girth enhancement was discussed with the patients. Written and verbal informed consent was obtained, including possible risks, complications, benefits, and alternatives, such as no surgery or an inflatable penile prosthesis. All patients chose to undergo Himplant® implantation which was performed between May 2022 through October 2022 through a high scrotal incision.

Intussusception of the penis through the lateral scrotal incision was more difficult due to prior degloving of the penis during surgical plication for Cases 1 and 2, and due to prior Xiaflex® injection therapy for Cases 2 and 3. As a result, adhesions between the underlying Dartos and Buck fascia were taken down sharply and great care was made to avoid thermal injury to the Dartos and skin side of the dissection. Such intricate dissection not only necessitates a high level of surgical dexterity but is also crucial for the successful insertion of Himplant®.

### Himplant® Specifications

The Himplant® is a penile prosthesis made of medical-grade silicone that is implanted subcutaneously through a high scrotal incision along the penile shaft (Fig. 1D) [16]. Its wall thickness ranges longitudinally from 1.5 to 2.5 cm, and it is offered in three lengths: 14, 16, and 18 cm. All three patients in this study received the 16 cm Himplant® (Fig. 1E).

Patients were monitored postoperatively weekly for 2 months postoperatively and every 2–3 months thereafter. Patients were asked to complete the International Index of Erectile Function (IIEF), a 15-question, 5-scale measure of erectile function, orgasmic function, sexual desire, intercourse satisfaction, and overall satisfaction [17]. Written consent was obtained prior to the procedure. Additionally, patients were asked the following questions with corresponding responses:

1. What is your satisfaction with the implant? (Very high, high, medium, low, very low)
2. How satisfied are you regarding penile girth enhancement with Himplant®? (Very high, high, medium, low, very low)
3. How satisfied are you regarding correction of penile curvature? (Very high, high, medium, low, very low, N/A).

## Results

The main findings of the study were successful correction of residual penile deformity (curvature, indentation, narrowing) and improvement in penile girth in all three cases following the placement of the Himplant® subcutaneous silicone penile prosthesis (Fig. 1F, G, Tables 1–2). The mean increase in flaccid dorsal length of the visible penis was 1.5 cm and the mean increase in flaccid midshaft girth was 2.0 cm (Table 2).

**Table 1:** Pre-operative data.

Case	Age	Penile Deformity				Penile Measurements (cm)		Previous Non-surgical Treatment History	Previous Surgical History
		Curvature	Indentation	Hinge	Narrowing	Flaccid Dorsal Length	Flaccid Midshaft Girth		
1	54	None	None	None	Yes	9.5	10.5	None	TAP with extra-tunical grafting (2021)
2	64	40 degrees dorsal & 20 degrees left	Left lateral	None	Yes	7.6	9.5	Xiaflex x3 (2014–2015)	TAP (2016)
3	63	30 degrees dorsal & mild right	None	None	Yes	10.2	9.9	Xiaflex x4 (2018)	None

**Table 2:** Post-operative data.

Case	Age	Penile Deformity				Penile Measurements (cm)		Complications
		Curvature	Indentation	Hinge	Narrowing	Flaccid Dorsal Length^a^	Flaccid Midshaft Girth^b^	
1	54	None	None	None	None	11	12	Seroma x2
2	64	None	None	None	None	9	11.8	Seroma
3	63	None	None	None	None	11.8	12.1	None

^a^Mean Δ = +1.5 cm (±0.1).

^b^Mean Δ = +2.0 cm (±0.4).

The patients were followed monthly for 14–19 months. Once permitted to engage in sexual intercourse, all patients reported that they were sexually active and engaging in penetrative sex. High patient satisfaction was demonstrated through both the IIEF and a 3-question, non-validated postoperative survey (Table 3). Mean IIEF scores, measured on a five-point scale for erectile function, orgasmic function, sexual desire, intercourse satisfaction, and overall satisfaction, were 29.67, 10, 9, 12.33, and 9, respectively. These scores closely align with data collected from healthy volunteers [17]. Additionally, the non-validated survey revealed high or very high satisfaction levels regarding the implant, girth enhancement, and correction of penile curvature across all three cases (Table 3). There was no new onset of ED. All patients reported normal penile function, including normal sensation, erection, orgasm, ejaculation, and urination, during the follow-up period. No patient reported loss of erect penile length. Although aspiration of seroma in the clinic was necessary twice for Patient 1 and once for Patient 2, no other complications have been observed in 19 months of follow-up.

**Table 3:** IIEF and non-validated patient satisfaction questionnaire data.

Case #	Date Collected	International Index Of Erectile Function (IIEF)					Unvalidated Satisfaction Survey		
		Erectile Function	Orgasmic Function	Sexual Desire	Intercourse Satisfaction	Overall Satisfaction	What is your satisfaction with your implant?	How satisfied are you regarding girth enhancement with Himplant?	How satisfied are you regarding correction of penile curvature?
1	11/21/23	30	10	10	13	8	Very High	High	N/A
2	11/29/23	29	10	9	11	9	High	High	Very High
3	11/21/23	30	10	8	13	10	Very High	Very High	Very High

## Discussion

There is no reliable surgical or non-surgical treatment for PD that can restore the penis to its pre-PD state. A variety of options are currently used in an effort to make the penis functionally straight and preserve or enhance its rigidity. Men who have residual bothersome deformity after initial treatment with intralesional Xiaflex® and plication procedures are not uncommon [18, 19].

Treatment options have fallen short of complete correction in men with PD who can experience devastation from the changes in his erect penis. We believe that men who have been disappointed with the results of attempts to correct their deformity with contemporary treatment options may be suitable candidates for placement of a Himplant®. Candidates are men with stable PD, satisfactory erectile function with or without PDE-5i oral therapy, and who have curvature <40° and indentation or narrowing, which does not result in instability or buckling during penetrative sex (often referred to as a hinge-effect).

Strengths of this study include meticulous patient selection, the utilization of the validated IIEF for multidimensional assessment of ED, and the achievement of positive functional and aesthetic results as perceived by the patients after Himplant®. In addition to having their perceived deformities corrected, they gained flaccid girth and length which is the primary goal for the Himplant®.

Limitations of this study include the small cohort size, limiting the conclusiveness of the findings, and the relatively short follow-up period. The use of a 3-item non-validated questionnaire is descriptive so patient responses may not be reliable. Additionally, the lack of pre- and post-operative photographs for cases 1 and 3 constrains the visual assessment of surgical results in these cases. Finally, the study highlights the need for a standardized evaluation technique for penile deformity pre- and post-intervention, which is currently lacking [20, 21].

## Conclusion

The objective of this perspective article is to present Himplant® as a novel corrective option for bothered patients experiencing residual penile deformities, such as curvature, indentation, or narrowing, following contemporary treatment for PD. Based on this small case series, Himplant® placement was shown to be effective for three patients in correcting mild residual deformities with respect to curvature, indentation, and/or narrowing following previous PD treatment of Xiaflex® injections and/or plication surgery. Additional longitudinal studies with larger cohorts are required to further validate the efficacy, safety, and long-term outcomes of Himplant® in patients with residual deformity following treatment for PD, as well as to assess the durability of the corrective results.

## Data Availability

All data generated or analyzed during this study are included in this published article and its supplementary information files.
